# Challenges in upscaling laboratory studies to ecosystems in soil microbiology research

**DOI:** 10.1111/gcb.16537

**Published:** 2022-11-28

**Authors:** Ji Chen, Yong Zhang, Yakov Kuzyakov, Dong Wang, Jørgen Eivind Olesen

**Affiliations:** ^1^ State Key Laboratory of Loess and Quaternary Geology Institute of Earth Environment, Chinese Academy of Sciences Xi'an China; ^2^ Department of Agroecology Aarhus University Tjele Denmark; ^3^ iCLIMATE Interdisciplinary Centre for Climate Change Aarhus University Roskilde Denmark; ^4^ Key Laboratory of Soil Ecology and Health in Universities of Yunnan Province, School of Ecology and Environmental Sciences, Yunnan University Kunming China; ^5^ Department of Soil Science of Temperate Ecosystems University of Göttingen Göttingen Germany; ^6^ International Joint Research Laboratory of Global Change Ecology School of Life Sciences, Henan University Kaifeng China; ^7^ Aarhus University Centre for Circular Bioeconomy, Aarhus University Tjele Denmark

**Keywords:** field in situ observation, global change factors, laboratory incubation, microbial‐based models, soil biogeochemistry, soil microbiology

## Abstract

Soil microbiology has entered into the big data era, but the challenges in bridging laboratory‐, field‐, and model‐based studies of ecosystem functions still remain. Indeed, the limitation of factors in laboratory experiments disregards interactions of a broad range of in situ environmental drivers leading to frequent contradictions between laboratory‐ and field‐based studies, which may consequently mislead model development and projections. Upscaling soil microbiology research from laboratory to ecosystems represents one of the grand challenges facing environmental scientists, but with great potential to inform policymakers toward climate‐smart and resource‐efficient ecosystems. The upscaling is not only a scale problem, but also requires disentangling functional relationships and processes on each level. We point to three potential reasons for the gaps between laboratory‐ and field‐based studies (i.e., spatiotemporal dynamics, sampling disturbances, and plant–soil–microbial feedbacks), and three key issues of caution when bridging observations and model predictions (i.e., across‐scale effect, complex‐process coupling, and multi‐factor regulation). Field‐based studies only cover a limited range of environmental variation that must be supplemented by laboratory and mesocosm manipulative studies when revealing the underlying mechanisms. The knowledge gaps in upscaling soil microbiology from laboratory to ecosystems should motivate interdisciplinary collaboration across experimental, observational, theoretic, and modeling research.

## Graphical abstract

The rapid technological advancements for high‐throughput sequencing and omics analyses have enabled soil microbiology to enter into a big data era across the ever‐increasing temporal and spatial scales. However, there are significant dilemmas when bridging laboratory‐, field‐, and model‐based data sources for an advanced understanding of soil microbiology. We discussed the reasons for the dilemmas and proposed several suggestions for the future research priorities. These dilemmas should motivate interdisciplinary collaboration across experimental, observational, theoretic, and modeling research.

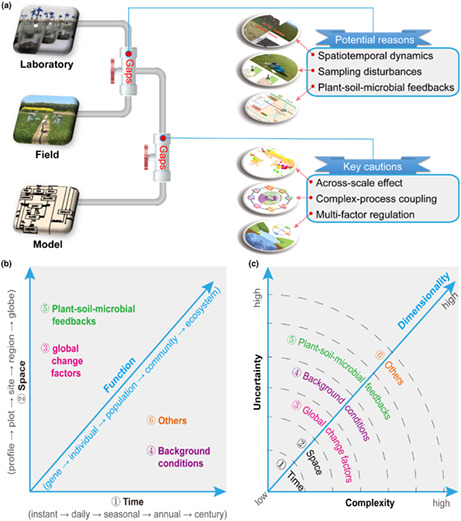

The rapid technological advancements for high‐throughput sequencing and omics analyses have enabled soil microbiology to enter into a big data era across the ever‐increasing temporal and spatial scales (Chen & Sinsabaugh, 2021; Lui et al., 2021; Xia et al., 2020). Such fast data accumulation has profoundly advanced the understanding of changes in soil microbial communities under various environment conditions as well as their ecological functions (Luo et al., 2016; Smercina et al., 2021). For example, emerging studies provide compelling evidence that explicit incorporation of microbial processes into mechanistic modeling has substantially improved projections of soil carbon (C) and nutrient cycling and reduced model uncertainties (Wang, Gao, et al., 2021; Wieder et al., 2015). These studies have spurred laboratory‐scale and field‐scale research to explore the patterns and mechanisms of soil microbial communities, functions, and the ecological implications. Laboratory and field studies have their separate advantages and disadvantages, whereas they differ greatly in boundary conditions, for example, drivers, scales, complexity, and particularly soil structure and the interactions with plants (Lui et al., 2021; Standing et al., 2007). Therefore, findings from laboratory‐ and field‐based studies do not always agree and sometimes are even contradictory (Feng et al., 2017; Jian et al., 2020). The disagreements between laboratory‐ and field‐based studies have been the major challenges when upscaling these studies to regional and global scales. However, these challenges are not adequately considered in many current models, likely misleading model projections.

The disagreement between laboratory‐ and field‐based studies is observed when incorporating N‐cycling microbial guilds to predict soil N_2_O emission, despite increasing studies showed that soil microorganisms play crucial roles in modulating soil N_2_O emission in both laboratory‐ and field‐based studies (Shi et al., 2021; Zhang et al., 2022). Based on globally field‐based studies, Zhang et al. (2022) show that N addition significantly raised both N‐cycling microbial guild abundances and soil N_2_O emission but without clear causal relationships between them. On the contrary, close correlations between N‐cycling microbial guild abundances and soil N_2_O emission are observed in laboratory‐ and mesocosm‐based studies (Shi et al., 2021). Such discrepancies suggest that solely laboratory‐informed model frameworks may overestimate the microbial contribution to in situ changes of soil N_2_O emission. One explanation is the underrepresentation of key environmental factors in the laboratory‐based studies (Zhang et al., 2022), for example, precipitation, soil pH, soil C:N, and ecosystem type, including the interactions with plants.

Challenges are documented for microbially mediated soil C cycling when integrating laboratory‐, field‐ and model‐based studies. By optimizing microbial parameters based on short‐term laboratory‐incubations, Jian et al. (2020) predicted soil organic carbon (SOC) losses by 8% with warming. Their modeling results, however, are not supported by field‐based studies of unchanged SOC (van Gestel et al., 2018). Furthermore, Jian et al. (2020) even predicted a minor SOC gain by 2% when calibrating microbial parameters against long‐term laboratory incubations, contrasting to the model predictions based on short‐term laboratory incubations. Consequently, the study duration can be an important issue to bridge laboratory, field and modeling studies. For example, field‐based 26‐year observations from the Havard forest showed clearly that the long‐term responses of soil respiration to warming are distinct from the short‐term observations (Melillo et al., 2017).

The dilemmas between laboratory‐, field‐, and model‐based studies are existed regarding soil phosphorus (P) cycling. Based on the Liebigs law of minimum, many laboratory‐based studies predicted an increased plant and microbial P limitation after the enhanced atmospheric N deposition (Luo et al., 2022). On the contrary, a global meta‐analysis of 668 field‐based observations worldwide showed that enhanced N addition significantly increased P limitation in the short term, whereas long‐term continuous N addition might not necessarily aggregate P limitation (Chen et al., 2020). One explanation is that N‐induced P limitation in field‐based studies is progressively alleviated in the long term through the initial stimulation of soil microbial metabolic activity, soil phosphatase activity and plant–soil‐microbial feedbacks, thereby securing P supply to support plant and microbial growth (Chen et al., 2020). Without including the field‐based plant–soil‐microbial feedbacks, Earth System Models project increased P limitation with N addition, which would likely turn ecosystems from net CO_2_ sinks to net sources. However, by considering the in situ plant–soil–microbial feedbacks, Fleischer et al. (2019) showed that ecosystems can continuously serve as net CO_2_ sinks with long‐term N addition. Indeed, the challenges in bridging laboratory‐, field‐, and model‐based studies are widely observed in soil microbiology but are just starting to be recognized, which constitute major challenges for upscaling inferences and providing reliable evidence for policymaking.

There are three causes for the misalignment between laboratory‐ and field‐based studies (Figure 1): (1) Spatiotemporal dynamics. Laboratory studies are conducted under well‐controlled conditions, which are distinct from field studies, especially when considering the large diurnal, seasonal and annual variations, and stochastic events of many environmental factors (Feng et al., 2017; Jian et al., 2020). (2) Sampling disturbances. Soils used for laboratory studies are highly disturbed and homogenized (e.g., sieving), which may accelerate the release of occluded resources and nutrients (Feng et al., 2017). Specifically, the mixing of hotspots with low‐activity areas likely leads to underestimation of microbial metabolic functions, despite their non‐linearity. (3) Plant–soil–microbial feedbacks. Absence of external resource inputs in many laboratory‐based studies causes fast drop of microbial metabolic activities and process rates, likely resulting in cascading but understudied feedbacks between plants and microbes (Mariotte et al., 2018). For example, by investigating soil heterotrophic respiration from 110 field dryland observations across the global, Ye et al. (2019) showed that soil heterotrophic respiration was best predicted by incorporating a positive relationship between CUE and temperature, whereas laboratory‐based studies showing reductions in CUE with increasing temperature (Wang, Qu, et al., 2021).

**FIGURE 1:**
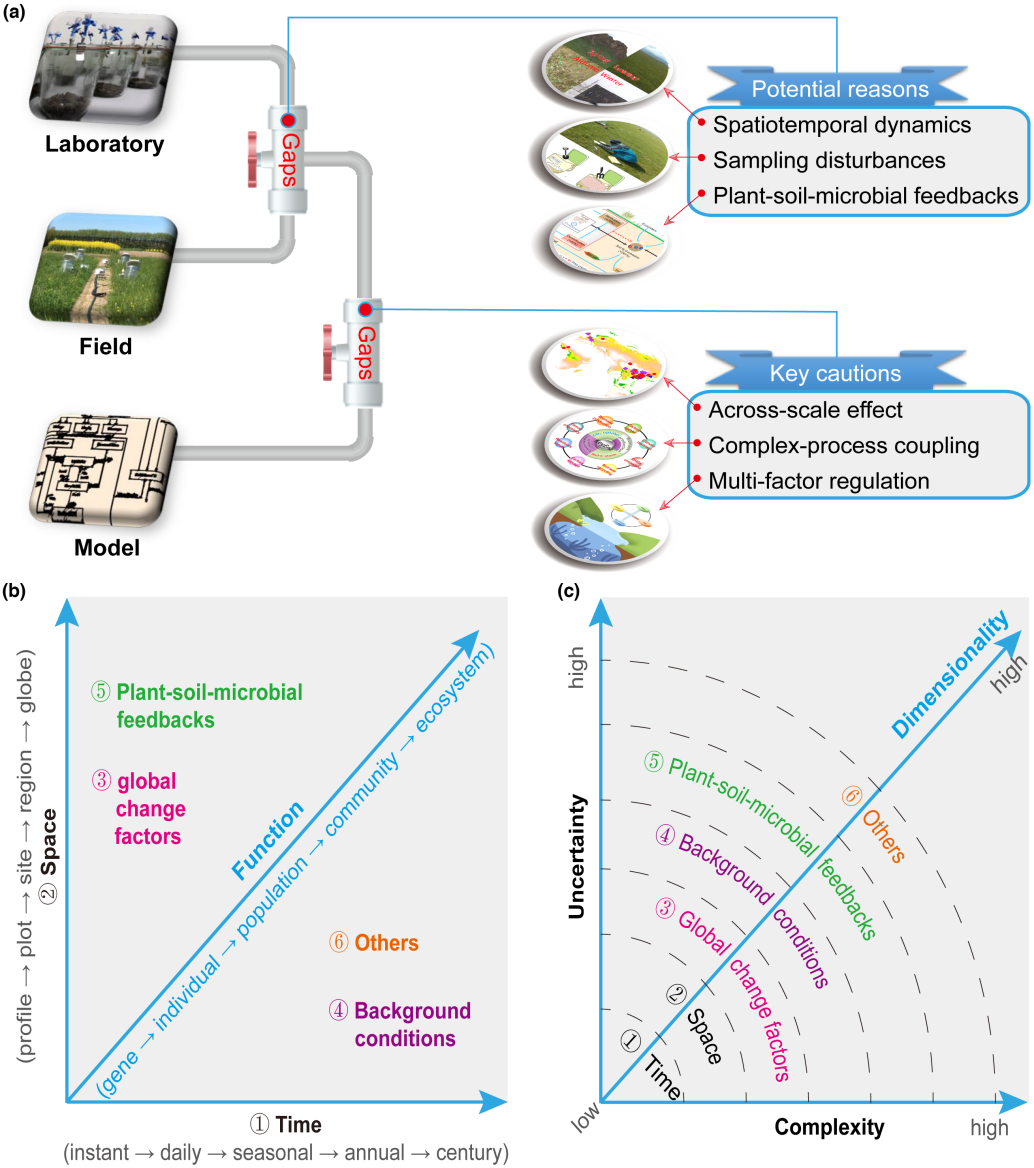
Challenges and reasons in bridging laboratory‐, field‐, and model‐based studies in soil microbiology research.

Caution is required in bridging knowledge gained from controlled environment studies with ecosystem scale studies and integrating this knowledge into models that can credibly predict effects of changing environmental conditions at relevant ecosystem scales (Figure 1). First, the across‐scale effect concerns how the underlying processes and associated mechanisms differ across spatial and temporal scales. For example, processes investigated on a small scale may be unimportant at ecosystem scale, for example, because the processes studied are overshadowed by other determinants such as those influenced by broad‐scale environmental variables. Second, complex‐process coupling issues may result from applying results of short‐term observational studies to long‐term situations due to the underrepresentation of factors affecting microbial adaptation and acclimation. For example, Jian et al. (2020) predicted contrasting effects of warming on soil C stock, when model parameters were tuned by either short‐ or long‐term studies. Third, multi‐factor regulation concerns microbial activities jointly, whereas the separate effects from various biotic and abiotic factors are difficult to disentangle (Matchado et al., 2021; Standing et al., 2007). It becomes even more challenging when considering the impacts of multiple global change drivers, since the present field‐based manipulation studies are mostly conducted under a single factor.

Despite the dilemmas in bridging laboratory‐ and field‐based studies, there are their own advantages in advancing the understanding of soil microbiology (Lui et al., 2021). Laboratory‐based studies can effectively explore microbial sensitivities and the underlying mechanisms to experimental factors under well‐controlled conditions, which can be strong to advance the mechanistic understanding. Field‐based studies can capture the environmental and climatic variations and plant–soil feedbacks, which can better reflect the entire ecosystem responses. However, field‐based observations often cover a limited range of environmental conditions that must be supplemented by laboratory or mesocosm studies if we want to advance the mechanistic understanding. For example, to explore the temperature sensitivity of soil respiration, laboratory studies offer considerable control on soil moisture under various temperatures, whereas the side effects of warming on soil moisture cannot be avoided in field‐based experiments even under a minor warming magnitude (Feng et al., 2017). Laboratory‐based studies can help reveal underlying processes and mechanisms that are underrepresented in the current model frameworks, although the associated constraints should be fully considered when integrating data from different sources. Therefore, future research should seek effective methods to resolve the dilemmas in bridging laboratory‐, field‐, and model‐based studies and better integrate data from different sources.

Scientists are developing tools to integrate these studies across scales for more accurate understanding and predictions, despite the fundamental gaps between laboratory‐, field‐, and model‐based studies. An example is data assimilation, in which model frameworks are iteratively modified by incorporating new data and mechanisms to fit the latest observations, although such data integration is only appropriate if the data cover the range of interest. For example, Chen et al. (2019) developed a data‐driven enzyme model using data assimilation based on the new relationships between soil extracellular enzymes and SOC dynamics as recently reported by another meta‐analysis (Chen et al., 2018). This data assimilation approach can well simulate the reported relationships between enzymes and SOC, which significantly improved the model projections of SOC dynamics under enhanced N addition at Duke Forests. To better resolve these challenges, we propose to strengthen the following research areas to inform the microbial traits‐based frameworks (Figure 2). (1) Improved observational networks (e.g., global changes, biodiversity, C–N fluxes) under diverse environmental conditions, despite it is the least of inspiration if at the first glance. This will allow models representing new mechanistic understanding to be tested under a range of diverse real‐life conditions. It can be achieved by building on existing broad‐scale network observations and long‐term ecosystem experiments (Wieder et al., 2015). (2) Exploring useful proxies (e.g., genes, enzymes, and other functional traits) for the species‐rich soil microbial communities (Treseder et al., 2012; Trivedi et al., 2013). For example, by representing various C‐N‐associated enzyme‐mediated processes into the Microbial‐Enzyme Decomposition model as a proxy of microbial processes, Wang, Gao, et al. (2021) profoundly improved model simulations of soil C and N cycling. (3) Applying emerging advanced data‐analytic approaches (e.g., hierarchical random‐matrix, eco‐evolutionary dynamics, machine learning, ecological networks) for integrating the mechanistic understanding of key microbial processes with data from multiple scales (Matchado et al., 2021; Zhang et al., 2022). This will enable us to reveal the new mechanisms among mounting biotic and abiotic variables, which are not possible to be discovered using the conventional methods. Despite these suggestions cannot be exhaustively complete in bridging laboratory‐, field‐, and model‐based studies, the strengthened interdisciplinary collaborations across experimental, observational, theoretic, and modeling research are highly warranted.

**FIGURE 2:**
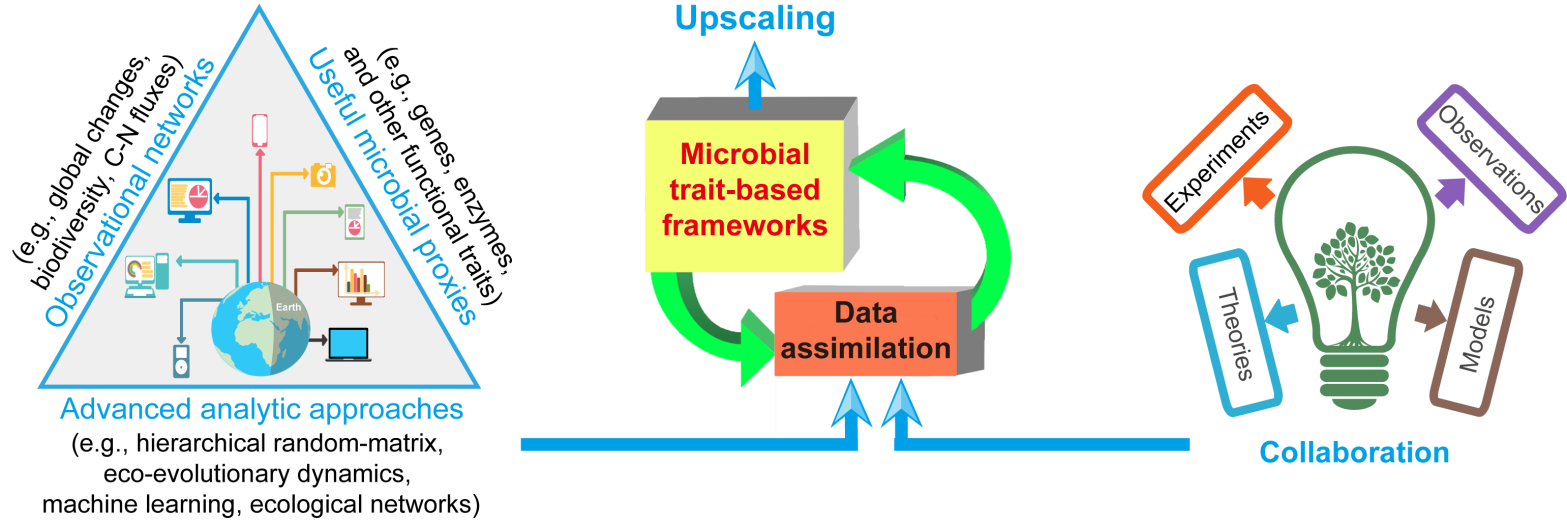
Future efforts that may help address current challenges in upscaling laboratory studies to ecosystems in soil microbiology research.

## AUTHOR CONTRIBUTIONS

J.C., Y.Z., Y.K., D.W. and J.E.O. contributed equally to this paper.

## CONFLICT OF INTEREST

The authors declare no conflict of interest.

## Data Availability

Data sharing is not applicable to this article as no new data were created or analyzed in this study.

## References

[gcb16537-bib-0001] Chen, J. , Luo, Y. , Van Groenigen, K. J. , Hungate, B. A. , Cao, J. , Zhou, X. , & Wang, R.‐W. (2018). A keystone microbial enzyme for nitrogen control of soil carbon storage. Science. Advances, 4(8), eaaq1689. 10.1126/sciadv.aaq1689 PMC6105232 30140736

[gcb16537-bib-0002] Chen, J. , & Sinsabaugh, R. L. (2021). Linking microbial functional gene abundance and soil extracellular enzyme activity: Implications for soil carbon dynamics. Global Change Biology, 27, 1322–1325. 10.1111/gcb.15506 33372345

[gcb16537-bib-0003] Chen, J. , Van Groenigen, K. J. , Hungate, B. A. , Terrer, C. , Van Groenigen, J.‐W. , Maestre, F. T. , Ying, S. C. , Luo, Y. , Jørgensen, U. , Sinsabaugh, R. L. , Olesen, J. E. , & Elsgaard, L. (2020). Long‐term nitrogen loading alleviates phosphorus limitation in terrestrial ecosystems. Global Change Biology, 26(9), 5077–5086. 10.1111/gcb.15218 32529708

[gcb16537-bib-0004] Chen, Y. , Chen, J. , & Luo, Y. (2019). Data‐driven ENZYme (DENZY) model represents soil organic carbon dynamics in forests impacted by nitrogen deposition. Soil Biology and Biochemistry, 138, 107575. 10.1016/j.soilbio.2019.107575

[gcb16537-bib-0005] Feng, W. , Liang, J. , Hale, L. E. , Jung, C. G. , Chen, J. , Zhou, J. , Xu, M. , Yuan, M. , Wu, L. , Bracho, R. , Pegoraro, E. , EAG, S. , & Bracho, R. (2017). Enhanced decomposition of stable soil organic carbon and microbial catabolic potentials by long‐term field warming. Global Change Biology, 23(11), 4765–4776. 10.1111/gcb.13755 28597589

[gcb16537-bib-0006] Fleischer, K. , Rammig, A. , De Kauwe, M. G. , Walker, A. P. , Domingues, T. F. , Fuchslueger, L. , Garcia, S. , Goll, D. S. , Grandis, A. , Jiang, M. , Haverd, V. , Hofhansl, F. , Holm, J. A. , Kruijt, B. , Leung, F. , Medlyn, B. E. , Mercado, L. M. , Norby, R. J. , Pak, B. , … Lapola, D. M. (2019). Amazon forest response to CO_2_ fertilization dependent on plant phosphorus acquisition. Nature Geoscience, 12(9), 736–741. 10.1038/s41561-019-0404-9

[gcb16537-bib-0007] Jian, S. , Li, J. , Wang, G. , Kluber, L. A. , Schadt, C. W. , Liang, J. , & Mayes, M. A. (2020). Multi‐year incubation experiments boost confidence in model projections of long‐term soil carbon dynamics. Nature Communications, 11(1), 5864. 10.1038/s41467-020-19428-y PMC7672078 33203846

[gcb16537-bib-0008] Lui, L. M. , Majumder, E. L. W. , Smith, H. J. , Carlson, H. K. , Von Netzer, F. , Fields, M. W. , Stahl, D. A. , Zhou, J. , Hazen, T. C. , Baliga, N. S. , Adams, P. D. , & Arkin, A. P. (2021). Mechanism across scales: A holistic modeling framework integrating laboratory and field studies for microbial ecology. Frontiers in Microbiology, 12, 642422. 10.3389/fmicb.2021.642422 33841364 PMC8024649

[gcb16537-bib-0009] Luo, M. , Moorhead, D. L. , Ochoa‐Hueso, R. , Mueller, C. W. , Ying, S. C. , & Chen, J. (2022). Nitrogen loading enhances phosphorus limitation in terrestrial ecosystems with implications for soil carbon cycling. Functional Ecology, 36, 2845–2858. 10.1111/1365-2435.14178

[gcb16537-bib-0010] Luo, Y. , Ahlström, A. , Allison, S. D. , Batjes, N. H. , Brovkin, V. , Carvalhais, N. , Chappell, A. , Ciais, P. , Davidson, E. A. , Finzi, A. , Georgiou, K. , Guenet, B. , Hararuk, O. , Harden, J. W. , He, Y. , Hopkins, F. , Jiang, L. , Koven, C. , Jackson, R. B. , … Zhou, T. (2016). Toward more realistic projections of soil carbon dynamics by earth system models. Global Biogeochemical Cycles, 30(1), 40–56. 10.1002/2015GB005239

[gcb16537-bib-0011] Mariotte, P. , Mehrabi, Z. , Bezemer, T. M. , De Deyn, G. B. , Kulmatiski, A. , Drigo, B. , Veen, G. F. C. , van der Heijden, M. G. A. , & Kardol, P. (2018). Plant–soil feedback: Bridging natural and agricultural sciences. Trends in Ecology & Evolution, 33(2), 129–142. 10.1016/j.tree.2017.11.005 29241940

[gcb16537-bib-0012] Matchado, M. S. , Lauber, M. , Reitmeier, S. , Kacprowski, T. , Baumbach, J. , Haller, D. , & List, M. (2021). Network analysis methods for studying microbial communities: A mini review. Computational and Structural Biotechnology Journal, 19, 2687–2698. 10.1016/j.csbj.2021.05.001 34093985 PMC8131268

[gcb16537-bib-0013] Melillo, J. M. , Frey, S. D. , Deangelis, K. M. , Werner, W. J. , Bernard, M. J. , Bowles, F. , Pold, G. , Knorr, M. A. , & Grandy, A. (2017). Long‐term pattern and magnitude of soil carbon feedback to the climate system in a warming world. Science, 358(6359), 101–105. 10.1126/science.aan2874 28983050

[gcb16537-bib-0014] Shi, Y. , Wang, J. , Ao, Y. , Han, J. , Guo, Z. , Liu, X. , Zhang, J. , Mu, C. , & Le Roux, X. (2021). Responses of soil N_2_O emissions and their abiotic and biotic drivers to altered rainfall regimes and co‐occurring wet N deposition in a semi‐arid grassland. Global Change Biology, 27(19), 4894–4908. 10.1111/gcb.15792 34240513

[gcb16537-bib-0015] Smercina, D. N. , Bailey, V. L. , & Hofmockel, K. S. (2021). Micro on a macroscale: Relating microbial‐scale soil processes to global ecosystem function. FEMS Microbiology Ecology, 97(7), fiab091. 10.1093/femsec/fiab091 34223869

[gcb16537-bib-0016] Standing, D. , Baggs, E. M. , Wattenbach, M. , Smith, P. , & Killham, K. (2007). Meeting the challenge of scaling up processes in the plant–soil–microbe system. Biology and Fertility of Soils, 44(2), 245–257. 10.1007/s00374-007-0249-z

[gcb16537-bib-0017] Treseder, K. K. , Balser, T. C. , Bradford, M. A. , Brodie, E. L. , Dubinsky, E. A. , Eviner, V. T. , Hofmockel, K. S. , Lennon, J. T. , Levine, U. Y. , MacGregor, B. J. , Pett‐Ridge, J. , & Waldrop, M. P. (2012). Integrating microbial ecology into ecosystem models: Challenges and priorities. Biogeochemistry, 109(1), 7–18. 10.1007/s10533-011-9636-5

[gcb16537-bib-0018] Trivedi, P. , Anderson, I. C. , & Singh, B. K. (2013). Microbial modulators of soil carbon storage: Integrating genomic and metabolic knowledge for global prediction. Trends in Microbiology, 21(12), 641–651. 10.1016/j.tim.2013.09.005 24139848

[gcb16537-bib-0019] van Gestel, N. , Shi, Z. , Van Groenigen, K. J. , Osenberg, C. W. , Andresen, L. C. , Dukes, J. S. , Hovenden, M. J. , Luo, Y. , Michelsen, A. , Pendall, E. , Reich, P. B. , EAG, S. , & Hungate, B. A. (2018). Predicting soil carbon loss with warming. Nature, 554(7693), E4–E5. 10.1038/nature25745 29469098

[gcb16537-bib-0020] Wang, C. , Qu, L. , Yang, L. , Liu, D. , Morrissey, E. , Miao, R. , Liu, Z. , Wang, Q. , Fang, Y. , & Bai, E. (2021). Large‐scale importance of microbial carbon use efficiency and necromass to soil organic carbon. Global Change Biology, 27(10), 2039–2048. 10.1111/gcb.15550 33559308

[gcb16537-bib-0021] Wang, G. , Gao, Q. , Yang, Y. , Hobbie, S. E. , Reich, P. B. , & Zhou, J. (2021). Soil enzymes as indicators of soil function: A step toward greater realism in microbial ecological modeling. Global Change Biology, 28(5), 1935–1950. 10.1111/gcb.16036 34905647

[gcb16537-bib-0022] Wieder, W. R. , Allison, S. D. , Davidson, E. A. , Georgiou, K. , Hararuk, O. , He, Y. , Hopkins, F. , Luo, Y. , Smith, M. J. , Sulman, B. , Todd‐Brown, K. , Wang, Y.‐P. , Xia, J. , & Xu, X. (2015). Explicitly representing soil microbial processes in earth system models. Global Biogeochemical Cycles, 29(10), 1782–1800. 10.1002/2015GB005188

[gcb16537-bib-0023] Xia, J. , Wang, J. , & Niu, S. (2020). Research challenges and opportunities for using big data in global change biology. Global Change Biology, 26(11), 6040–6061. 10.1111/gcb.15317 32799353

[gcb16537-bib-0024] Ye, J. S. , Bradford, M. A. , Dacal, M. , Maestre, F. T. , & García‐Palacios, P. (2019). Increasing microbial carbon use efficiency with warming predicts soil heterotrophic respiration globally. Global Change Biology, 25(10), 3354–3364. 10.1111/gcb.14738 31216082

[gcb16537-bib-0025] Zhang, Y. , Zhang, F. , Abalos, D. , Luo, Y. , Hui, D. , Hungate, B. A. , García‐Palacios, P. , Kuzyakov, Y. , Olesen, J. E. , Jørgensen, U. , & Chen, J. (2022). Stimulation of ammonia oxidizer and denitrifier abundances by nitrogen loading: Poor predictability for increased soil N_2_O emission. Global Change Biology, 28(6), 2158–2168. 10.1111/gcb.16042 34923712 PMC9303726

